# Utility of Excision and Direct Closure in Burns: A Case Report and Review of the Literature

**DOI:** 10.7759/cureus.79455

**Published:** 2025-02-22

**Authors:** Nhan S Trieu, Jonathan Butts, Peter Vonu, Shawn Larson, Kalyan Dadireddy

**Affiliations:** 1 Plastic and Reconstructive Surgery, University of Florida, Gainesville, USA; 2 Pediatric Surgery, University of Florida, Gainesville, USA; 3 Plastic Surgery, University of Florida, Gainesville, USA

**Keywords:** body contouring, breast burns, breast reduction surgery (brs), burn treatment, direct closure

## Abstract

In the past years, the mainstay of burn treatment has been early tangential excision and skin grafting. Excision with direct closure is another reconstructive approach that has not been extensively explored. This study aims to review the existing literature on the utility of excision and direct closure, including prospective series, case series, correspondence, and randomized controlled trials. We also present our case report of a 12-year-old patient who sustained self-inflicted burns totaling 35% total body surface area (TBSA) and underwent bilateral mammaplasty, consistent with the excision and direct closure technique.

The study finds that direct closure of burn wounds requires sufficient tissue laxity to allow tension-free closure. This technique has multiple advantages, including reduced burn surface area, decreased rates of hypermetabolism and infections, quicker healing time, shorter hospital stays, lower healthcare costs, and better esthetic outcomes. In our patient's case, her bilateral breast burns were initially treated with tangential excision and allograft, which were complicated by poor graft adhesion and pseudomonal colonization. Subsequently, the plastic surgery team excised burned breast tissues using the principles of reduction mammaplasty with superomedial-central pedicle to mobilize the remaining healthy breast parenchyma and skin to create an esthetic breast mound. No additional breast surgery was needed during the patient’s remaining hospital stay.

In summary, while having similar rates of postoperative complications to traditional burn treatment, excision and direct closure offer several advantages. However, primary closure may not be suitable for patients with an extensive burn surface area without an adequate tissue reservoir. Therefore, this technique should be further investigated as the treatment option for a selective burn population.

## Introduction

Compared to early tangential excision and skin grafting, excision with direct closure offers an alternative approach to deep burn reconstruction, provided there is sufficient laxity around the wound [1,2]. Though it has never gained the same popularity as the current mainstay in burn treatments, this method was successfully performed as far back as 1929, particularly for treating small, deep burn areas [3]. In 1985, Gahhos et al. conducted pioneering work by performing a series of direct burn closures or closures using local flaps. In their case series, eight out of nine patients showed no evidence of hypertrophic scar formation and only one patient developed a 4-cm dehiscence on a large back burn, which healed with conservative treatments [2].

Despite the potential benefits of quicker healing times and shorter hospital stays, the excision and direct closure technique faces significant reluctance among burn surgeons, primarily due to the concerns of scar hypertrophy and wound dehiscence. Nevertheless, there is currently no literature that supports these apprehensions [1,4]. In a prospective series by Bain et al., which included 100 burn patients, the median healing time for patients undergoing direct closure with partial skin grafting was twice as long as those undergoing direct closure alone. The dehiscence rate was 13.5% for partial graft and 10.8% for direct closure. Hypertrophic scars were present in 10.8% of burns in both treatment groups [1]. Another group of surgeons associated with three burn centers in the Netherlands performed a randomized controlled trial that compared the short-term and long-term outcomes of acute burn patients undergoing split-thickness skin graft versus primary closure using a skin-stretching device. This study demonstrated that while there were no significant differences in short-term complications or long-term appearance between the two methods, patients in the skin-stretching and direct closure group had a significant reduction in the surface area of burn scars [5].

Our study conducts an extensive literature review of the utility of burn wound excision and direct closure. We further aim to present a case report of a 12-year-old patient who sustained bilateral breast full-thickness burns and underwent reduction mammaplasty based on the principles of excision and direct closure.

This article was previously presented as an oral presentation at the SESPRS 67th Annual Scientific Medical Student Meeting on April 26, 2024, and as a visual abstract at the 2024 University of Florida Surgery Research Day on May 9, 2024.

## Case presentation

A 12-year-old female patient is evaluated after sustaining self-inflicted trauma involving rubbing alcohol and fire, resulting in 35% total body surface area (TBSA) of partial to full-thickness burns to her face, neck, chest, abdomen, and extremities. She was initially treated with tangential excision followed by allograft placement. However, in the month following surgery, chest wound healing was complicated by her large breast volume, which led to difficulties with dressing management, poor allograft adhesion, and topical pseudomonal growth.

Given that the self-immolation was triggered by teasing about her large breasts, and in light of the wound complications, plastic surgery was consulted. After extensive consent and psychological evaluation, the decision was made to perform excision and direct closure in the form of bilateral reduction mammoplasty to alleviate the wound and dressing burden. Despite the presence of pseudomonal sloughing, the procedure was deemed necessary as it would allow for the removal of the infected tissue and reduce breast size and weight, improving healing by facilitating better dressing management. This second procedure was performed six weeks after the initial surgery.

**Figure 1:**
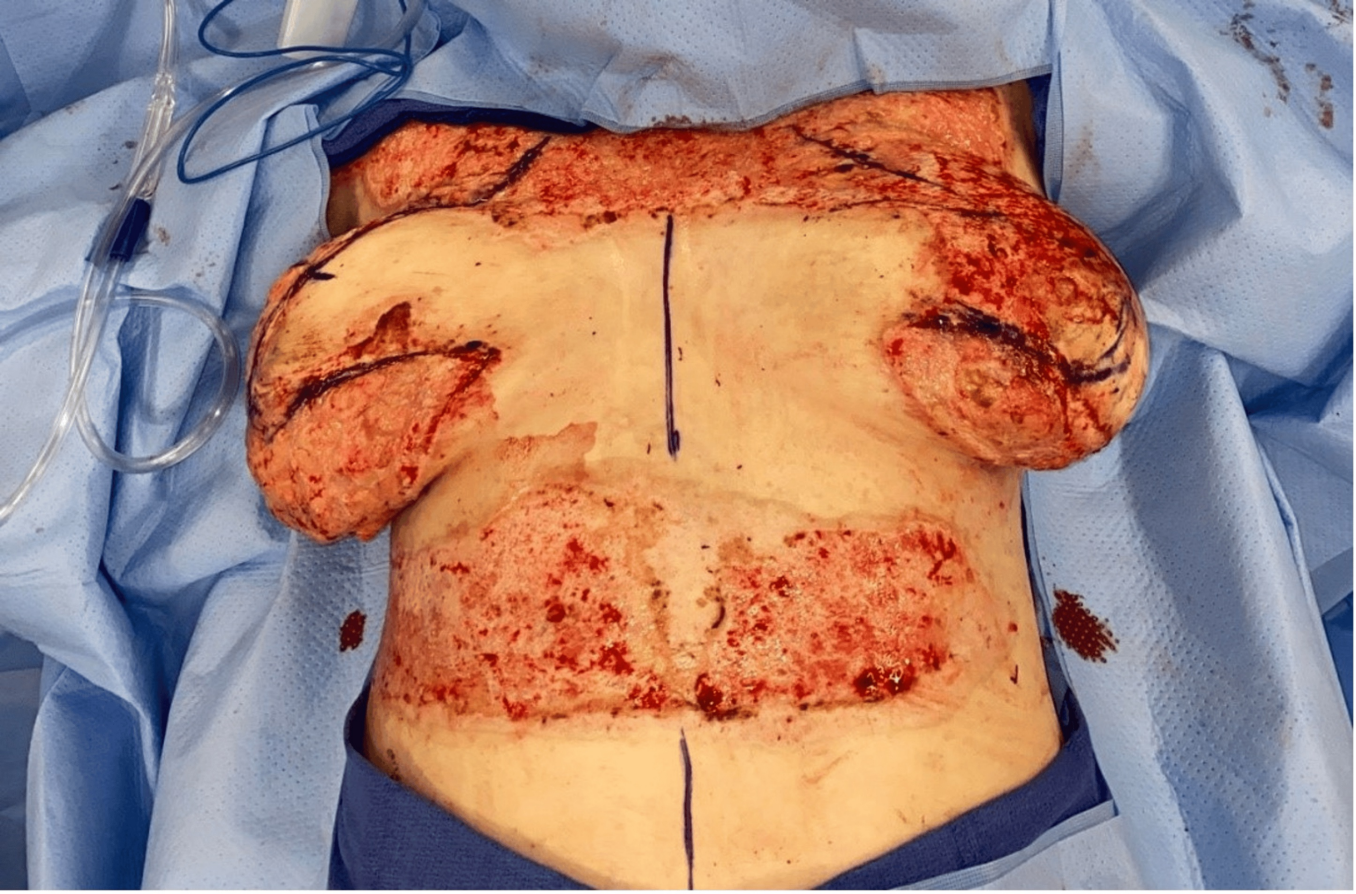
Preoperative markings

**Figure 2:**
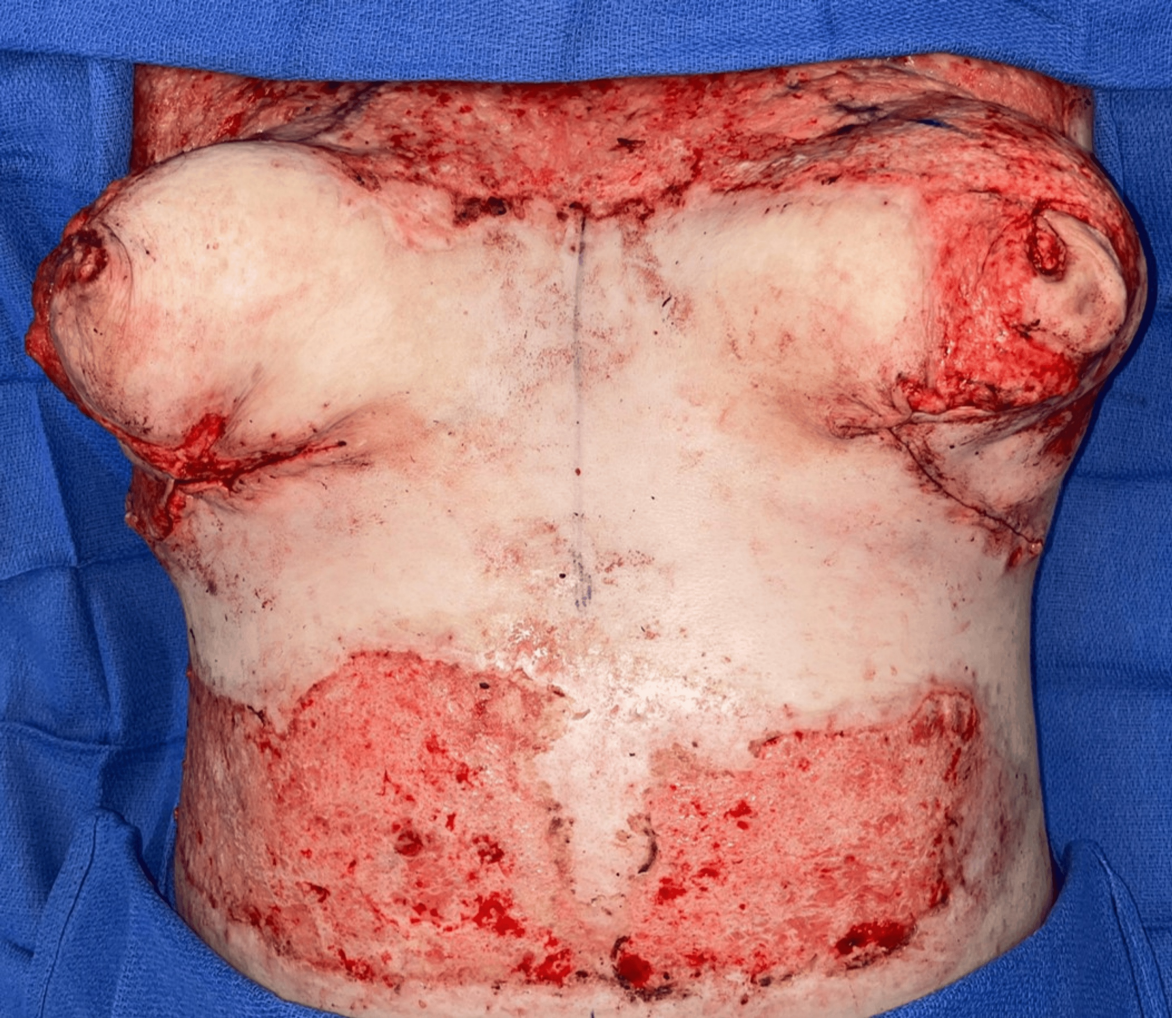
Immediate postoperative outcome

The procedure was executed using the superomedial-central pedicle technique in which the anterior branches of intercostal perforators were preferentially preserved along with the superomedial pedicle to maximize nipple-areolar complex (NAC) perfusion, leading to less reliance on dermal blood supply. Areas of burned tissues removed included inferomedial, inferior, and inferolateral breast tissues and additional parenchyma 5 cm below the NAC (Figure 1). The medial and lateral breast pillars were approximated to stabilize the breast form and optimize shape and projection. To reduce the chance of infections, monofilament sutures, Polydioxanone (PDS, Ethicon, Somerville, NJ, US) and Monocryl (Ethicon, Somerville, NJ, US), are used for parenchymal and deep dermal closure, respectively. At the completion of closure, the nipple complexes were pink and well vascularized (Figure 2).

**Figure 3:**
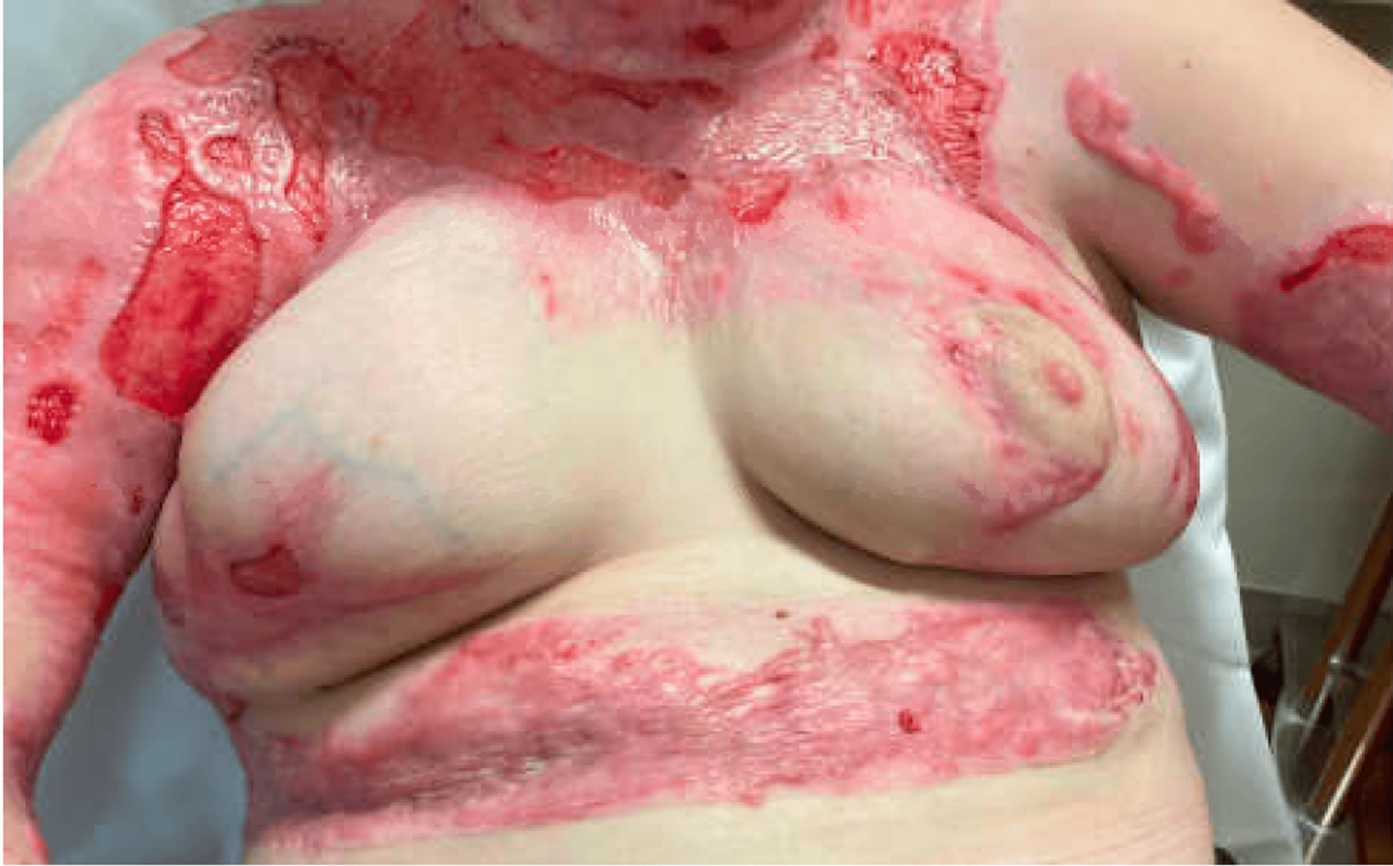
Bilateral breast postoperative week 15

The postoperative course was notable for the right breast incisional dehiscence along the lateral pillar that was treated conservatively. The patient’s breasts healed extremely well with a minor deficiency to the right inferior pole, which would be amendable to fat grafting in the future. However, no additional breast surgery was needed during the patient’s remaining hospital stay. Her bilateral NAC continued to be well perfused and healed as expected. Given the extent of her chest burn and the significant amount of breast tissue removed, the surgery resulted in some distortion of anatomical landmarks, as well as potential contracture and widened scars. Despite these challenges, the bilateral breast reduction not only alleviated the wound and dressing burden for the primary care team but also provided significant relief for our patient’s psychological trauma. The patient returned to our outpatient clinic four months after her breast reduction procedure. Physical examination at that time was notable for scar contracture, causing lateral deviation of the right NAC (Figure 3). Otherwise, she was reportedly very pleased with the result of the mammoplasty.

## Discussion

Management of burns has significantly advanced in recent decades, yet the use of excision and direct closure remains relatively unexplored and underutilized [1,4]. Using this technique, the plastic and reconstructive team successfully reduced the areas of breast burn, sparing the patient from lengthy additional skin grafting. The procedure was made possible by the large breast volume the patient had before her injury, which provided sufficient tissue laxity. The approach was particularly beneficial in this 12-year-old patient with large breasts, which were both a source of concern and a contributing factor to her burn injury. Bilateral breast reduction as a treatment for burn wounds could also be a valuable option for women of all ages with excess breast tissue, offering an alternative to traditional skin grafting.

Direct skin closure also played a major role in infection control for the patient. In large TBSA burn cases, infections account for up to 75% of mortality and pose a substantial burden on the primary care team [6]. In this case, since pseudomonal growth was only found topically on wound sloughing, excision of burned tissues helped remove the source of infection and prevent further systemic spread. By primarily closing the burn wound, the plastic surgery team aimed to limit wound exposure to the outside environment, reducing the chance of additional infection. Moreover, the reduction of extensive burn areas on the chest alleviated the burden of wound care and dressing changes.

Candidates for excision and direct skin closure

The technique of direct closure is reserved for specific burn wounds with satisfactory adjacent tissue reservoirs [1,2,4,5]. All individuals with sufficient laxity, mainly of upper arms, breasts, upper chest, abdomen, and medial thighs, similar to those in body contouring patients, are ideal candidates [7]. This procedure can take place after initial resuscitation as the primary surgery, or at a later time such as in the case of our patient.

Initially proposed as a technique for smaller burn wounds, Gahhos et al. demonstrated the efficacy of direct closure in treating larger defects, including a 15 x 60-cm muffler burn, a 22 x 18-cm full-thickness burn that was closed in a V-Y fashion using two superiorly based flaps, and a 20 x 8-cm burn to the left breast [2]. While some sources did not limit this technique to a specific body part, others had determined that burn wounds to the lower limbs or wounds to any extremities that exceeded 33% of circumference were not as suitable for direct skin closure [4,5]. One explanation offered for this exclusion was perhaps the higher rate of dehiscence compared to the other body parts. However, this was not deemed as an absolute contraindication; rather, McKean et al. clarified that these wound areas, due to their “greater proportional wound dehiscence rates,” were not candidates for community follow-up [4]. Ultimately, one requirement suggested by all authors for this approach was the availability of sufficient tissue volume and laxity to allow successful tension-free closure [1,4]. In the randomized controlled trial conducted by Dutch surgeons, the skin-stretching device was used to provide the needed tissue relaxation [5]. The mechanical strain put on the skin when closing the wound under tension could not only promote dehiscence but also increase the differentiation of fibroblast to myofibroblast, thereby leading to scar hypertrophy [1,8].

Advantages of excision and direct skin closure

Primary excision and direct closure of acute burn wounds offer numerous advantages, making it a preferred strategy in specific cases. One of the biggest causes of mortality in burn patients is hypermetabolism, the systemic response to severe injury or inflammation. Early excision and closure of injured tissues are the single greatest strategy to combat this phenomenon [9,10]. We can reasonably deduce that comparable to closure using allograft, autograft, or other dermal substitutes, direct closure reduces the burn surface area and eliminates exposure of burn tissues to the element, thereby decreasing the chance of developing hypermetabolism. Moreover, this method can also contribute to the decreased rate of infection as it removes the portal entry to bacteria and uses healthy skin as a barrier for the wound [2]. Keeping in mind the paucity of literature on the topic, the direct closure approach has been shown to be a safe technique with a similar if not lower rate of complications than the traditional burn treatment [1,2,4,5].

When comparing patients undergoing direct closure and patients treated with skin grafting, data suggest significantly quicker healing time in the former group [1]. This does not account for the extensive treatment time patients in the skin-grafting group have to undergo to achieve successful allograft followed by autograft. This approach can be a viable treatment option, especially in cases involving small, scattered areas of deep burns or as part of more extensive burn injuries. Particularly observed in the context of industrial accidents in 1985 when direct closure was first proposed, workers exposed to molten metal or electrical contact who were treated with this approach were able to swiftly return to work [2]. Quicker healing time also translates to shorter hospital stays and lower healthcare costs. These cost-saving benefits, along with enhanced patient convenience, are more apparent when patients are able to follow up postoperatively with their primary care physicians instead of needing frequent visits with burn specialists [4].

Finally, we believe that direct skin closure offers superior esthetic outcomes for burn patients compared to the traditional treatment approach. This is primarily due to the significant reduction in the scar surface area while maintaining similar scar color and patient and observer scar assessment scores [5]. Contrary to the reason for the reluctance from most burn surgeons, direct skin closure is shown to result in a similar rate of hypertrophic scars as skin grafting. Bain et al. further argue that delayed healing, as seen in their skin grafting group, significantly increases the likelihood of hypertrophic scar formation [1]. Direct skin closure also eliminates the need for donor sites, which further contributes to the shortened healing time and the reduction of overall burn and graft donor wound size, resulting in better esthetic outcomes [1,5].

Disadvantages of excision and direct skin closure

Like other approaches to burns, this technique does not come without its disadvantages. Primarily, this strategy is not an appropriate intervention for wounds covering large surface areas with insufficient tissue laxity. Neither can we utilize it to treat burns to body parts that cannot afford to get rid of adequate subcutaneous tissue volume [1]. Limited data is available to compare the rate of dehiscence between conventional treatment and direct closure. Yet, one can reasonably argue that by bringing the edges of the burn together in the case of direct closure, there is a likelihood of increased tension with the tendency to pull the wound apart. Nevertheless, these dehiscent wounds, as seen in several studies, were noted to be small and superficial, which only required conservative treatment and healed within two weeks. There was one notable case of deep dehiscence that prompted a return to the operating room and subsequent skin grafting. Therefore, Bain et al. suggested that this rate of minor or superficial dehiscence is acceptable and does not warrant the rejection of direct closure for specific cases [1]. Another disadvantage of direct closure is the difficulty of determining the depth of necrosis in an acute setting, limiting the surgeon’s ability to expeditiously excise damaged tissue [2]. Lastly, this method is not suitable for patients with risk factors for wound healing complications, including diabetes, ischemic disease, active infection, and conditions associated with collagen or elastin abnormalities (e.g., Ehlers-Danlos syndrome) [1,4,5].

## Conclusions

The case of our 12-year-old patient demonstrates when excision with direct closure is the most optimal treatment approach for extensive burn injuries. Previously rejecting skin grafting, the patient’s bilateral breasts have met all the criteria for this alternative technique, including adequate tissue laxity, sufficient underlying tissue that can be removed, and a large burn area that can benefit from the reduction of burn surface area. These areas further pose concerns for poor wound beds due to adipose tissue and laxity, making excision and grafting difficult. As in our patient, direct closure can also be used once the patient is stabilized after initial excisions. While the complication rates are comparable to traditional burn treatments, direct closure provides additional esthetic effects for selected patient populations using breast and body contouring principles. Further research into excision and direct closure as a treatment for burn wounds could advance surgical burn care.
